# Electrically Tunable Nonlinearity at 3.2 Terahertz in Single-Layer Graphene

**DOI:** 10.1021/acsphotonics.3c00543

**Published:** 2023-08-14

**Authors:** Alessandra Di Gaspare, Osman Balci, Jincan Zhang, Adil Meersha, Sachin M. Shinde, Lianhe Li, A. Giles Davies, Edmund H. Linfield, Andrea C. Ferrari, Miriam S. Vitiello

**Affiliations:** † NEST, CNR—Istituto Nanoscienze and Scuola Normale Superiore, Piazza San Silvestro 12, Pisa 56127, Italy; ‡ Cambridge Graphene Centre, University of Cambridge, Cambridge CB3 0FA, U.K.; § School of Electronic and Electrical Engineering, University of Leeds, Leeds LS2 9JT, U.K.

**Keywords:** graphene, terahertz, nonlinearity, ionic liquid gate modulator

## Abstract

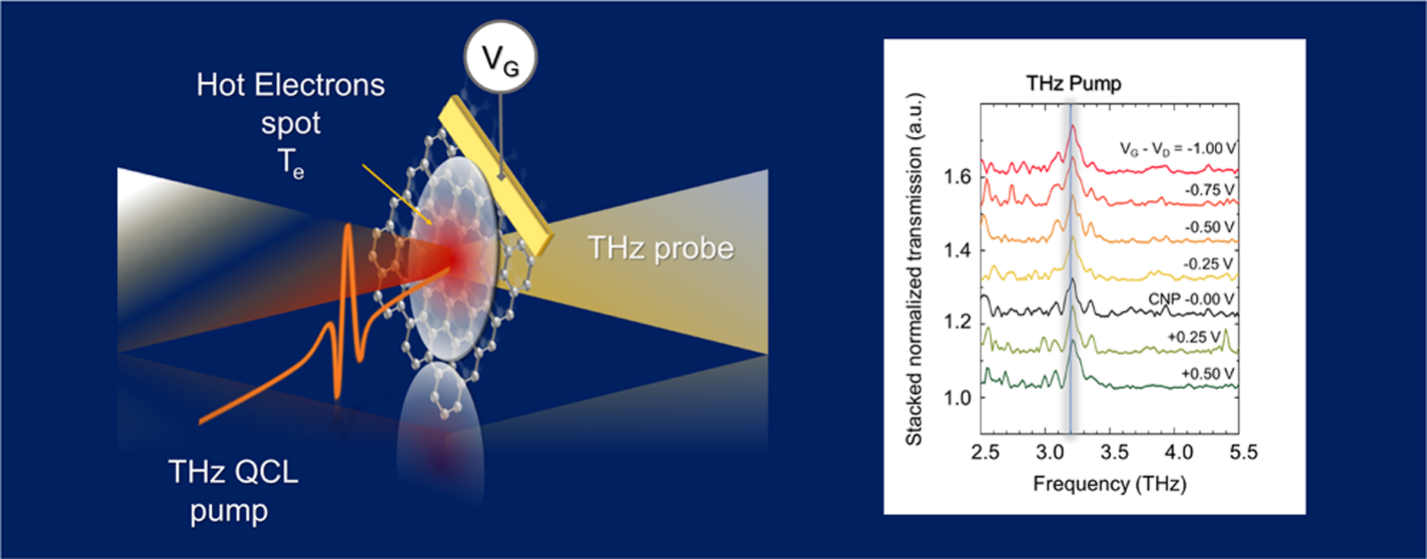

Graphene is a nonlinear material in the terahertz (THz) frequency range, with χ^(3)^ ∼ 10^–9^ m^2^/V^2^ ∼ 15 orders of magnitude higher than that of other materials used in the THz range, such as GaAs or lithium niobate. This nonlinear behavior, combined with ultrafast dynamic for excited carriers, proved to be essential for third harmonic generation in the sub-THz and low (<2.5 THz) THz range, using moderate (60 kV/cm) fields and at room temperature. Here, we show that, for monochromatic high peak power (1.8 W) input THz signals, emitted by a quantum cascade laser, the nonlinearity can be controlled using an ionic liquid gate that tunes the graphene Fermi energy up to >1.2 eV. Pump and probe experiments reveal an intense absorption nonlinearity at 3.2 THz, with a dominant 3rd-order contribution at *E*_F_ > 0.7 eV, hence opening intriguing perspectives per engineering novel architectures for light generation at frequencies > 9 THz.

## Introduction

Nonlinear optical devices, needed for ultrashort pulse generation,^1^ all-optical switching,^2,3^ frequency conversion and high harmonic generation (HHG),^4,5^ optical nanoimaging,^6^ energy harvesting,^7^ or for coherent control of quantum systems,^8^ require the integration of a highly nonlinear material in a core electronic or photonic device, with the possibility to control its nonlinear behavior. This usually requires altering the material absorption and refractive index through electronic effects,^9^ thermally induced effects,^10^ or through optical pumping with intense (hundreds kV/cm) electric fields.^11^

Atomic thin active layers offer the advantage to prevent dephasing and dispersion of the propagating wave, if compared with III–V semiconductors. Single-layer graphene (SLG) is a highly nonlinear material (χ^(3)^ ∼ 10^–9^ m^2^/V^2^ in the far-infrared)^12^ and it is thus ideal for integration into nonlinear optical devices.^13−15^ SLG has zero band gap and large electrical tunability, which shifts the Fermi energy *E*_F_, and induces Pauli blocking of the optical transitions.^16−18^ It also exhibits saturable absorption across a broad range of frequencies,^19−21^ large nonlinear optical response^12,22^ with a power transmission modulation of ∼ 50% per monolayer,^9−11,21^ ultrafast photoexcitation dynamics and recovery time,^22,23^ high chemical and mechanical stability,^24^ large thermal,^25^ and optical threshold damage,^26^ and it is also an extremely efficient THz frequency multiplier, with a huge 3rd-order nonlinearity,^27,28^ allowing for the direct generation of multiple harmonics in the 0.85–2.12 THz range.^27,28^

The strong optical nonlinearity^10,27,29^ and ultrafast dynamics^23^ have been exploited in far-infrared integrated optical modulators,^30^ saturable absorbers (SAs),^20,31^ exhibiting low saturation intensity (<6.7 W/cm^2^)^31^ and transparency modulations up to 80%,^31^ tunable SA mirrors,^31^ optical frequency combs,^32^ for driving semiconductor heterostructure lasers in the mode-locking regime,^33^ for engineering SAs for compact pulsed lasers in the microwave region,^34^ and for manipulating the polarization state of THz waves.^35^

At the low THz photon energies (4–12 meV), the SLG optical conductivity can be assumed to match the electrostatic one.^36−38^ This, in turn, depends on *E*_F_ and can be controlled through electrostatic gating,^37^ a property that discloses the potential of graphene for THz all-electronic reconfigurable modulators.^30^ Increasing the density of free carriers in SLG, and hence *E*_F_, enhances the power absorption of the THz driving field.^16^ Once the SLG carrier density is increased up to extremely high values (*n* > 10^14^ cm^–2^),^39,40^ it induces a strongly metallic-like behavior with a larger electronic heat capacity,^25^ which reduces the SLG thermodynamic nonlinearity, meaning that, at THz frequencies, an optimal *E*_F_ exists, which favors nonlinearity.

A variety of optically induced nonlinear phenomena such as HGG,^4^ four-wave mixing,^41^ and self-phase modulation^42^ via saturable photoexcited-carrier refraction have been reported in graphene, as an effect of single or multicycle driving fields with peak electric fields up to MV/cm and peak intensities up to TW/cm^2^ in the near-infrared.^4^ At low (≤2.15 THz) THz frequencies, the SLG nonlinearity, both for ultrashort single-cycle and quasi-monochromatic multicycle input signals, has been controlled using electrical gating and peak electric fields up to 80 kV/cm.^28^

Here, we investigate the nonlinear response of SLG at frequencies > 3 THz, exploiting an ionic liquid gate to tune *E*_F_. For low gate voltage (*V*_G_) (<1 V) and negligible leakage current (<100 nA/cm^2^), we reach *E*_F_ ∼ 1.2 eV, modulating the SLG broad-band linear optical conductivity in the THz range. The *E*_F_ dependence of the SLG absorption saturation is then assessed by means of open-aperture *z*-scan experiments. Pumping with a high-power (1.8 W) 3.2 THz quantum cascade laser (QCL), in a pump and probe configuration, reveals signatures of intense 3rd-order nonlinear absorption at 3.2 THz. We hence demonstrate the effects of *E*_F_ on SA and provide evidence of the associated nonlinear carrier dynamics in gated graphene, elucidating the role of hot electrons in eliciting thermal and field-driven 3rd-order nonlinearities in highly doped SLG. This may lead to the on-chip integration of nonlinear electrically pumped laser sources in unexplored frequency domains (6–10 THz).

## Results and Discussion

**Figure 1:**
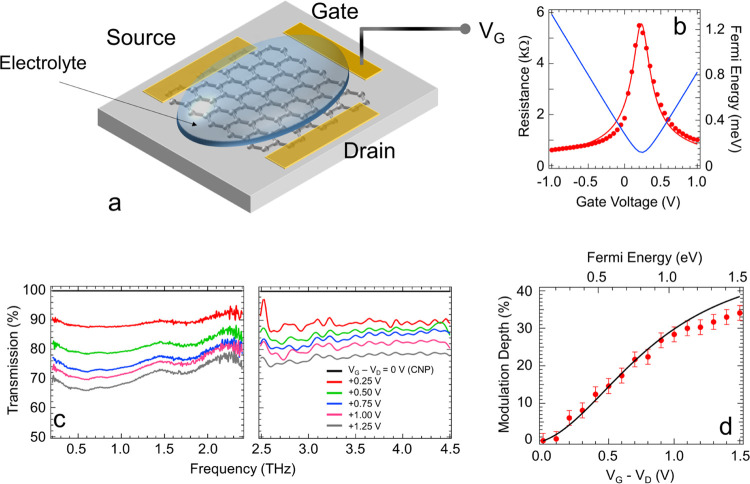
(a) Schematic diagram of the device: SLG is deposited on quartz, with source, drain, and gate electrodes. The ionic liquid electrolyte gate is employed to tune the SLG conductivity by applying *V*_G_ in steps of Δ*V* = +0.3 V. The transparent top quartz substrate contains the graphene system. (b) *V*_G_ dependence of resistance (red dots, left axis), compared with its fitted values (red line, left axis), extrapolated by employing the function
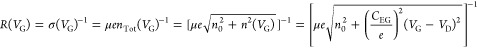
From the fit to the data, we extract a mobility μ of ∼ 1130 cm^2^/(V·s), a capacitance *C*_EG_ of ∼ 1.485 μF/cm^2^, and a residual carrier density *n*_0_ of ∼ 10^12^ cm^–2^. The *V*_G_ dependence of *E*_F_ (blue line, right axis) is extracted from:^36^, where v_F_ is the Fermi velocity of SLG, v_F_∼1.1·10^6^ m/s. (c) Transmission, measured at different *V*_G_/*E*_F_, by time domain spectrometer (TDS) in a N_2_-purged environment (humidity < 4%) to suppress atmospheric absorption lines and positioning the SLG device in the focus of a 50 mm focal length TPX lens (left), and by using an under-vacuum Fourier transform infrared spectroscopy (FTIR) spectrometer, with the SLG device placed at the focus of the FTIR internal compartment (right). Transmittance is retrieved by Fourier transform filtering of the transmission traces at each *V*_G_, then normalized to the curve retrieved at the charge neutrality point (CNP), corresponding to maximum transmission. Normalized transmission, measured at different *V*_G_/*E*_F_. (d) Modulation depth (MD) at different *V*_G_ (bottom axis) and *E*_F_ (top axis), retrieved by using the transmittance at 2 THz and the MD definition given in the main text (red dots); the solid line is MD from theoretical transmission as a function of σ, hence *E*_F_, defined in eq 1.

Figure 1 shows the device schematics. A SLG top-gate field effect transistor, exploiting an ionic liquid top gate,^43,44^ is fabricated on a 0.5 mm-thick quartz substrate, showing a 75% transparency in the THz range and no evidence of optically induced nonlinear effects.^21^ An electrolyte ionic liquid *N*,*N*-diethyl-*N*-methyl-*N*-(2-methoxyethyl ammonium-bis-trifluoromethanesulfonyl imide) of 99.9% has a large electrochemical power owing to the large surface electric field achievable^12^ (∼10–20 MV/cm), yielding a tunable *E*_F_ up to 1.2 eV.^45^ A self-forming ultrathin electrical double layer (EDL) at the SLG- and metal–electrolyte liquid interfaces generates electric fields of ∼10^9^ V/m at nanometer scales without electrical breakdown.^45^ The electrical double layer ensures high charge accumulation capability over a large area (∼mm^2^) with a negligible (∼pA) leakage current.^12,31,45^ An 8 × 8 mm^2^ SLG film is grown on Cu via chemical vapor deposition (CVD) and then transferred on the quartz substrate, using poly(methyl methacrylate) (PMMA)-assisted wet-transfer, as discussed in Materials and Methods. Source, drain, and gate metal contacts are then patterned using thermal evaporation with the aid of a shadow mask. The ionic gate is then sealed with another thin (0.1 mm) quartz substrate to prevent the liquid to spill out. The final device, operating in transmission mode, has an access optical window of ∼ 6 × 6 mm^2^ (see Materials and Methods).

Figure 1b shows the gate modulation of SLG resistance and *E*_F_. The conductivity has the typical ambipolar behavior, reaching a minimum at the charge neutrality point (CNP), *V*_D_ = +0.24 V. The residual conductivity stems from background charge carriers^36^ and is not altered by *V*_G_. By applying increasing *V*_G_ – *V*_D_, the carrier density varies, inducing a resistance modulation.

**Figure 2:**
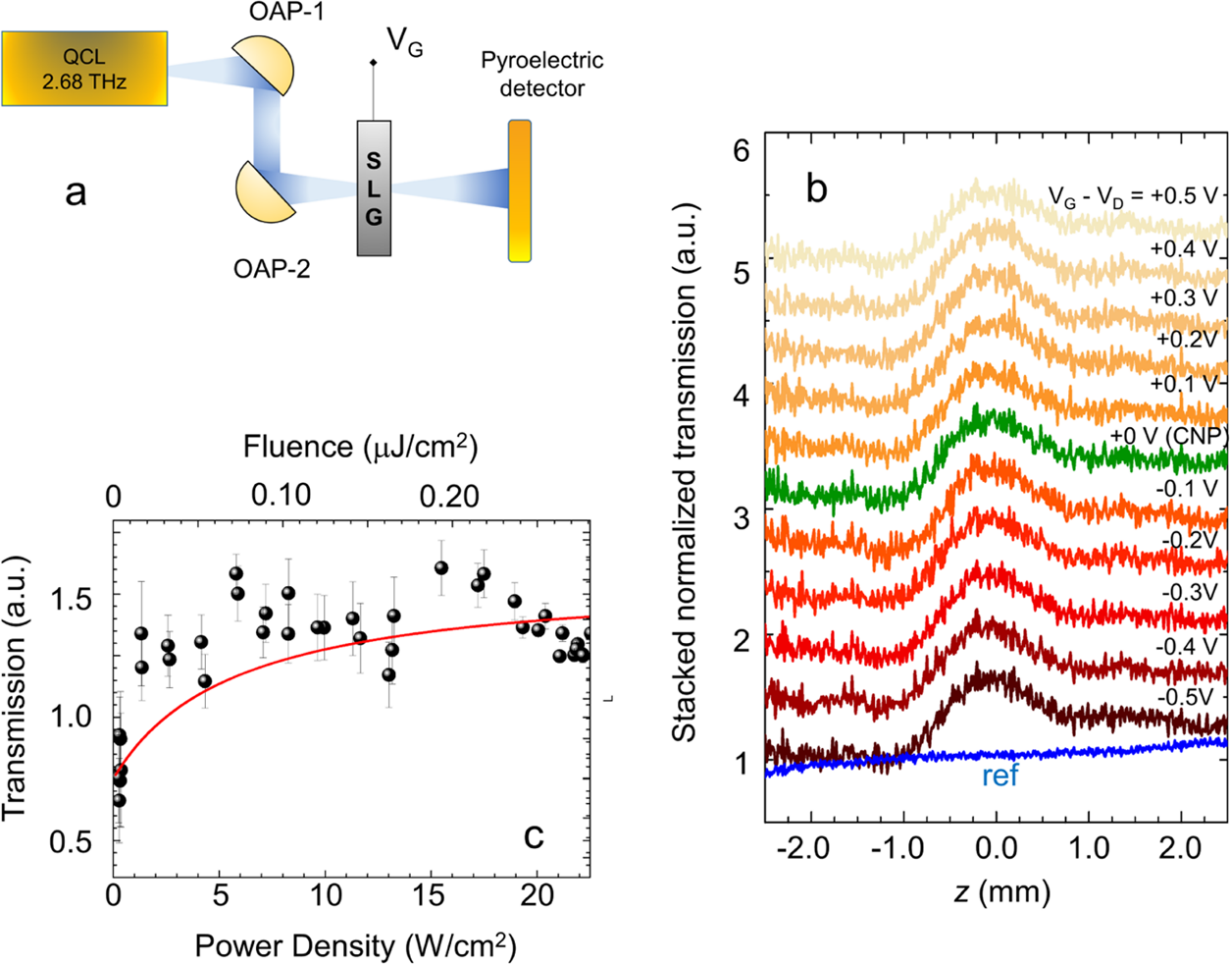
(a) Schematic diagram of the *z*-scan experiment. The device is mounted on a *z*–*y* linear stage moving along the optical beam axis (*z-*axis) and the perpendicular direction (*y* axis). The *y*, *z* maps are reconstructed by moving the stage around the focal point of the external cavity comprising two off-axis parabolic mirrors (OAPs) with a 50 mm focal length, and a 2.7 THz QCL at the opposite cavity focal point, emitting 6 mW. In the focal point (focus diameter 400 μm), the power density is ∼20 W/cm^2^. The transmitted signal is then collected by a pyroelectric detector positioned behind the SLG, measured by a lock-in amplifier as a function of the modulator (*y*, *z*) position and *V*_G_. (b) Stacked normalized transmission curves as a function of *z*, for individual *V*_G_, collected while measuring the signal transmitted by the modulator at different *V*_G_, and while changing *V*_G_ in steps of Δ*V*_G_ = ± |0.1| V around the CNP (green curve, *V*_G_ = +0.24 V) and then normalizing each curve with that collected on a reference sample, comprising the same substrate/ionic gate structure, but with no SLG on it. The blue line is the *z*-scan trace from the reference sample, showing no absorption enhancement around *z* = 0. The *z*-axis is shifted so that the modulator position *z* = 0 coincides with the focal plane. The *z*-scan traces are extracted by performing the integration of the transmitted signal also in the *y* direction (*y* scan range = 0.3 mm). (c) Enhanced transmission (black dots) as a function of the optical power intensity (bottom axis) and incoming fluence (top axis), defined as the energy density delivered on the sample in pulsed mode, *F* = *I* × *t*_pulse_, where *t*_pulse_ = 12.5 μs is the pulse duration, calculated as the peak normalized transmission (*z* = 0) in (b) at the CNP. The red line is the fit from eq 3, employed to extract the saturation intensity, *I*_S_.

The transmittance is measured using a purged time domain spectrometer (TDS) over the 0.1–2.5 THz range (Menlo System TeraK15). Each transmission trace (Figure 2d) *T*(*V*_G_) is normalized with that acquired at the CNP, revealing a broad-band modulation across the entire spectral range from 0.1 to >4.5 THz, reflecting the conductivity variation associated with *E*_F_ tuning. We then extract the modulation depth (Figure 1d) as:^46,47^. At 2 THz, MD > 30%, indicating that the device behaves as an efficient amplitude modulator. In the linear regime, the frequency-dependent optical transmission *T*(ν) at a given *V*_G_ is related to the *E*_F_-dependent SLG optical conductivity σ(ν) as^12^
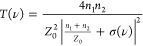
1where ν is the frequency, *Z*_0_ = 377 Ω is the free-space impedance, and *n*_1_ = 1.98 and *n*_2_ = 1.45 are the refractive indexes of the quartz substrate^48^ and of the ionic liquid gate,^49^ respectively. The *E*_F_-dependent σ(ν) comprises both interband, σ_inter_, and intraband, σ_intra_, terms. At THz frequencies, σ_inter_ is dominant, with a constant value of 2.3% per graphene layer, resulting in more than three orders of magnitude lower than σ_intra_.^36^ Consequently, it is here neglected. The total conductivity can be therefore calculated as^40^
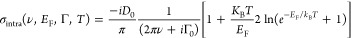
2where *D*_0_ = *E*_F_*e*^2^/ℏ^2^ is the linear Drude weight, *e* is the electron charge, *ℏ* is the reduced Planck constant, Γ_0_ = *ev*_F_^2^/*E*_F_ μ is the scattering rate, sensitive to *E*_F_ and to the carrier mobility μ, and *v*_F_ is the Fermi velocity. The broad-band optical response is slightly modulated (∼20%) by the frequency-dependent intraband conductivity, which, as expected, induces a weaker modulation at higher frequency,^17^ visible in Figure 1c, where the transmission modulation is higher in the <2 THz frequency range (left panel). In Figure 1d, we can compare the theoretical MD, calculated from eqs 1 and 2, with the experimental values. For *E*_F_ ≤ 1 eV, the experimental transmission modulation closely agrees with that calculated from the model. For *E*_F_ > 1 eV, the trend of the two curves remains in good agreement, but with the linear model exceeding the experimental maximum MD by ∼8%.^40^ Such a discrepancy, related to a decrease in the transmitted signal at higher *V*_G_, suggests a dynamic more complex than that explained by the aforementioned model when *E*_F_ > 1 eV, related to the dependence of μ from *E*_F_.^50^

To assess the nonlinear response of the SLG device, we perform an open-aperture *z*-scan experiment (see Figure 2a). The intensity of the electric field irradiating the sample is varied by exploiting the modulation of the optical power density in the focal region of a free-space optical cavity (Figure 2a), comprising two closely spaced off-axis parabolic mirrors having a 50 mm focal length, and a point-source in one cavity vertex. We use a single-plasmon 2.68 THz QCL, delivering 6 mW optical power, driven in pulsed mode with a pulse duration of 12.5 μs (repetition rate 40 kHz, duty cycle 50%).

The light absorption as a function of beam intensity is then measured by moving the sample within and outside the focal region at the opposite cavity vertex, scanning the position along the optical beam axis (*z*-scan) with a few μm-precision stage, and simultaneously collecting the transmitted light impinging on the 3 mm diameter optical window of a pyroelectric detector, positioned behind the focal plane at a distance set on purpose to allow the entire scanning of the focal region. To unveil the nonlinear behavior, we extract the transmittance modulation along the *z-*axis, around the focal region, by normalizing the traces acquired from the SLG device, as a function of *V*_G_, with that acquired on a reference device with an identical architecture but with no SLG (Figure 2b). A visible enhancement of the normalized transmission in proximity to the focal plane is retrieved. Its Lorentzian-like shape has a width dependent on the intensity profile across the beam waist. The transmission enhancement is slightly dependent on *V*_G_, as confirmed by the weak (<10%) variation of the peak transmittance retrieved at *z* = 0 for the traces obtained at different *E*_F_ in Figure 2b. The quantitative analysis of the absorption modulations as a function of *V*_G_ in Figure 2b is discussed in the following paragraphs in comparison with the outcome of the numerical theoretical model (Figure 4).

The observed increase of the transmission at the focal plane is a clear evidence of SA.^29,51^ It is a nonlinear process that depends on the impinging optical power and on the saturation intensity *I*_S_, that is, the intensity at which the SA is reduced by 50%.^20,31,40^ We then place the SLG, and then the reference sample, in the focal plane and retrieve the transmitted intensity as a function of the input power intensity. This is achieved by varying the QCL driving current in regular steps from the laser threshold to a regime in which the QCL emits a peak optical power of ∼6 mW. The position-dependent intensity *I*(*z*) = *I*_0_/(1 + (*z*/*z*_R_)^2^) can be written as a function of the beam intensity at the focal point *I*_0_ and of the Rayleigh length, *z*_R_, which expresses the distance, along the propagation direction of a beam, from the waist to the place where the area of the cross section is doubled and is extracted from a knife edge measurement that provides a circular spot size of 0.4 mm diameter. We then estimate *I*_S_ at the CNP by assuming that the normalized transmission intensity, as a function of the optical power intensity, *T*(*I*), follows the same dependence of the absorption coefficient on the pump intensity^17,20,52^ (see Figure 2c):
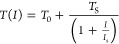
3where *T*_0_ is the unsaturated transmittance, measured at a *z* ∼ 2 mm, i.e., outside the focus region, and *T*_S_ is the maximum transmission enhancement at *z* = 0 (see the SI). Both *T*_0_ and *T*_S_ are extracted by normalizing the *z*-scan traces from the SLG and from the reference sample, hence *T*_0_ ≡ 1. From the fitting procedure (Figure 2c), we obtain *T*_S_ = 61 ± 6% and *I*_S_ = 5.63 ± 0.22 W/cm^2^ in agreement with previous reports.^20,31^

**Figure 3:**
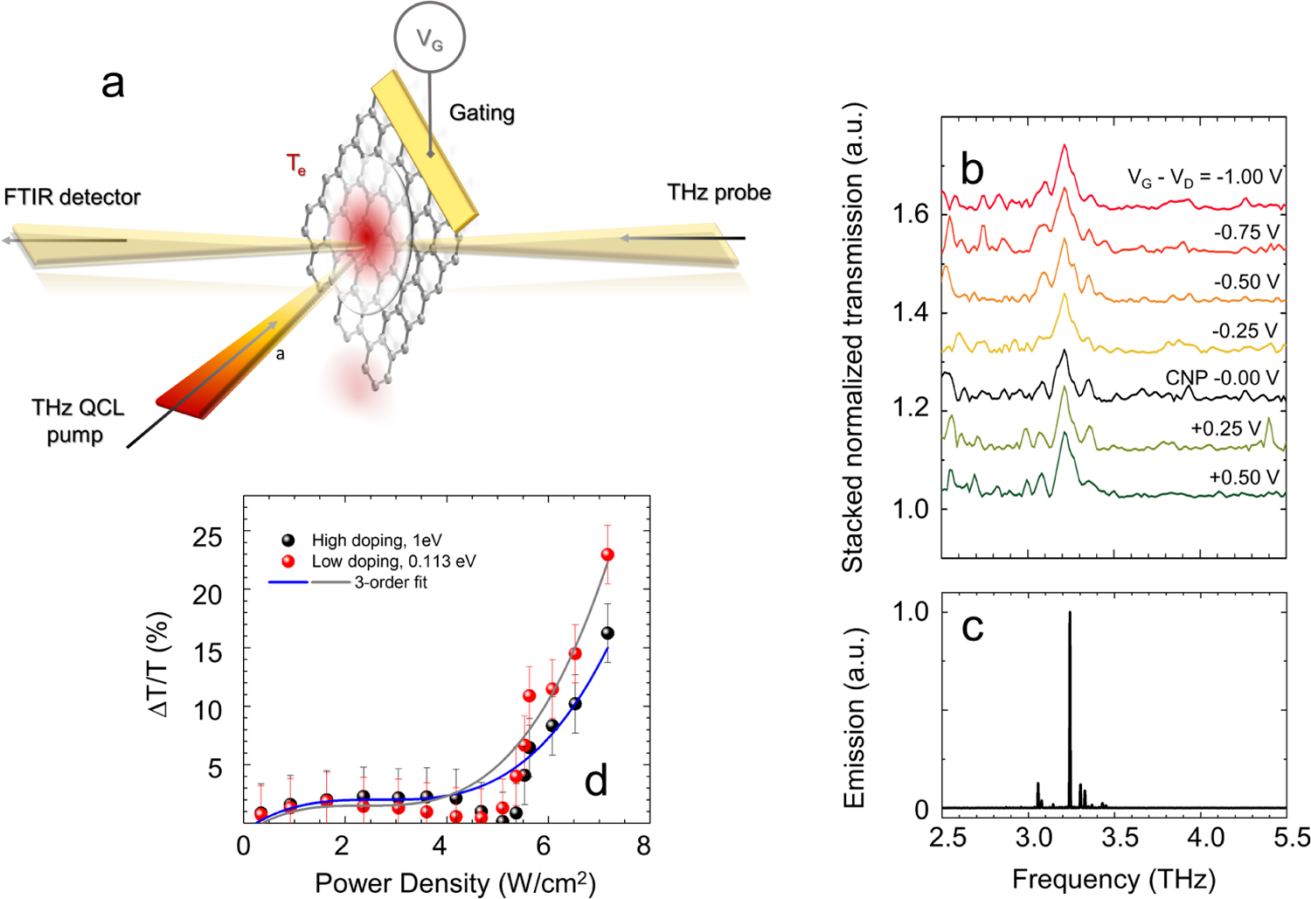
(a) Schematic diagram of the THz pump and probe measurement. (b) Stacked transmission curves retrieved from the ratio of the experimental transmissions measured on SLG (stacking step Δ*T* = 0.1), acquired while varying *V*_G_ with step Δ*V*_G_ = *V*_G_–*V*_D_ = 0.25 V when pumped with the QCL, *T*_ON_, and when the QCL is off, *T*_OFF_. The transmission is acquired under vacuum, in the sample compartment of an FTIR spectrometer (Bruker Vertex 80v) in rapid scan mode using a spectral resolution of 1 cm^–1^ and an aperture size of 2.5 mm. (c) FTIR emission spectrum of the single-plasmon QCL employed as a pumping source, driven in pulsed mode at 10 kHz with a pulse duration of 1 μs (duty cycle 1%) at a current *I* = 9.5 A, corresponding to a peak optical power impinging on a sample of ∼1.8 W, accounting for the reflection losses of the cryostat and of the spectrometer optical windows. (d) Experimental transmission change, Δ*T*/*T*, at the CNP (black dots) and at *E*_F_ = 1 eV (red dots), as a function of the pump QCL power density, measured in the setup shown in panel (a), while keeping the FTIR movable mirror at a fixed position. The QCL driver was amplitude-modulated with a 317 Hz TTL signal, while acquiring the signal, detected by a He-cooled Si-bolometer (Infrared Laboratory), with a lock-in amplifier. Δ*T*/*T* is extracted by subtracting the linear term, measured on the reference sample, to the signal retrieved from the SLG. The gray and blue curves are extracted by a 3rd-order polynomial fit (fit procedure correlation factor *R* = 0.94) of the traces retrieved at the CNP and *E*_F_ = 1 eV, respectively. To determine the order of the nonlinearity, we perform a set of polynomial fits, leaving the polynomial order as a free parameter, up the 4th order, and setting the lower-order polynomial terms to zero.

We then perform a pump and probe spectroscopic experiment to assess the nonlinear response of the SLG (Figure 3). For this purpose, we employ, as a pump, a THz frequency light pulse emitted by a high-power (∼2.5 W peak power) single-plasmon QCL. The pump excites the charge carriers in the SLG. The SLG spectral response is subsequently detected by measuring the transmission with a high-resolution under-vacuum FTIR spectrometer (Bruker, Vertex 80v). The QCL is focused on the sample, mounted inside the FTIR, by means of a set of off-axis parabolic mirrors (OAPs) and a lens through a THz-transparent (85% transmission) optical access window. The SLG is placed at the focal point of both the interferometric and the QCL beam paths, so that it is simultaneously illuminated by the broad-band FTIR internal source and by the pumping QCL. The sample positioning is then optimized to suppress the stray light coming from the pumping source that can eventually impinge on the detector. During this procedure, a reference sample is employed to rule out any modulation arising from the SLG, with the pump on. We first finely adjust the pump beam focus so that it matches the desired spot on the sample surface. We then optimize the incidence angle by suppressing the stray light contribution. The transmission curves, retrieved on the SLG sample, as a function of *V*_G_ (Figure 3b), unveil an increase of transmission when the sample is illuminated by the pump, in the same spectral range of the QCL emission (Figure 3c).

We calculate the pump-enhanced transmission (Figure 3d) by normalizing the spectral trace acquired at each *V*_G_ with the pump on, with the corresponding trace collected when the QCL is off. The peak transmission centered at ∼3.21 THz matches the center of the QCL emission band (Figure 3c). The transmission enhancement is <20% at the CNP, i.e., lower than that extracted from the *z*-scan experiment (≥60%), despite the significantly larger pumping intensity. This is partly an effect of the longer optical path and mainly the consequence of the fact that the detector signal is encoding both the pump on and off terms, with the on-term contributing only for a small portion of the pulse (1 μs pulse width at a percentage of the total integration time equal to 1% of the duty cycle employed).

To quantitatively elucidate the nonlinear effects, we then acquire the transmission signal by keeping the FTIR moving mirror at a fixed position and by varying the fluence of the pumping source by increasing the QCL driving current in regular steps following the same procedure of Figure 2c. We first focus the light on the reference sample, then on the SLG device, when it is at the CNP and at high |*V*_G_ – *V*_D_| = 1.0 V, *E*_F_ = 1 eV. We extract the transmission modulation, Δ*T*(*V*_G_)/*T* = [*S*_SLG_ (*V*_G_) – α*S*_ref_]/α*S*_ref_, where *S*_SLG_(*V*_G_) is the transmission acquired with the SLG at *V*_G_, *S*_ref_ is the signal from the reference sample, and α is a normalization parameter so that Δ*T*/*T* = 0 at the onset of the nonlinear behavior, accounting for the difference of signal magnitude from the two samples, in the linear regime (Figure 3c). No evidence of nonlinear response is seen up to the laser threshold, i.e., at a threshold intensity *I*_th_ ∼ 5.4 W/cm^2^, in agreement with the *I*_S_ value retrieved from the *z*-scan experiment. For *I* > *I*_th_, a nonlinear increase in the transmittance is visible. By fitting the experimental data with a polynomial curve (Figure 3d), we extract a 3rd-order power law for the transmission enhancement, in agreement with the SA theory of ref (29).

Both the *z*-scan and pump and probe experiments indicate a nonlinear response of the SLG device. The nonlinear interaction between free carriers in SLG and the field of the driving THz source resembles a thermodynamic process exhibiting an interplay of heating–cooling dynamics.^11,27^ In the THz range, the intraband absorption in SLG of an intense (∼10–20 kV/cm) optical beam leads to a nonequilibrium excess distributions of carriers at the energy of the optical pump. At first, the ultrafast (∼20 fs) carrier–carrier scattering^23,27^ drives the initial redistribution of the energy absorbed by the photons, leaving the system in a nonequilibrium state characterized by a hot electron distribution with electrons at temperature *T*_e_.^53^ Then, depending on the type of excitation and on the available cooling channels, the system relaxes to an equilibrium state.^53^ Here, owing to pulse duration in the μs scale, the optical excitation varies on a time scale much longer than that associated with the heating–cooling dynamics of the SLG carriers. This effectively realizes a steady excitation state at *T*_e_, where a nonlinear modulation of the SLG optical response can emerge, stemming from the temperature-dependent optical conductivity.

**Figure 4:**
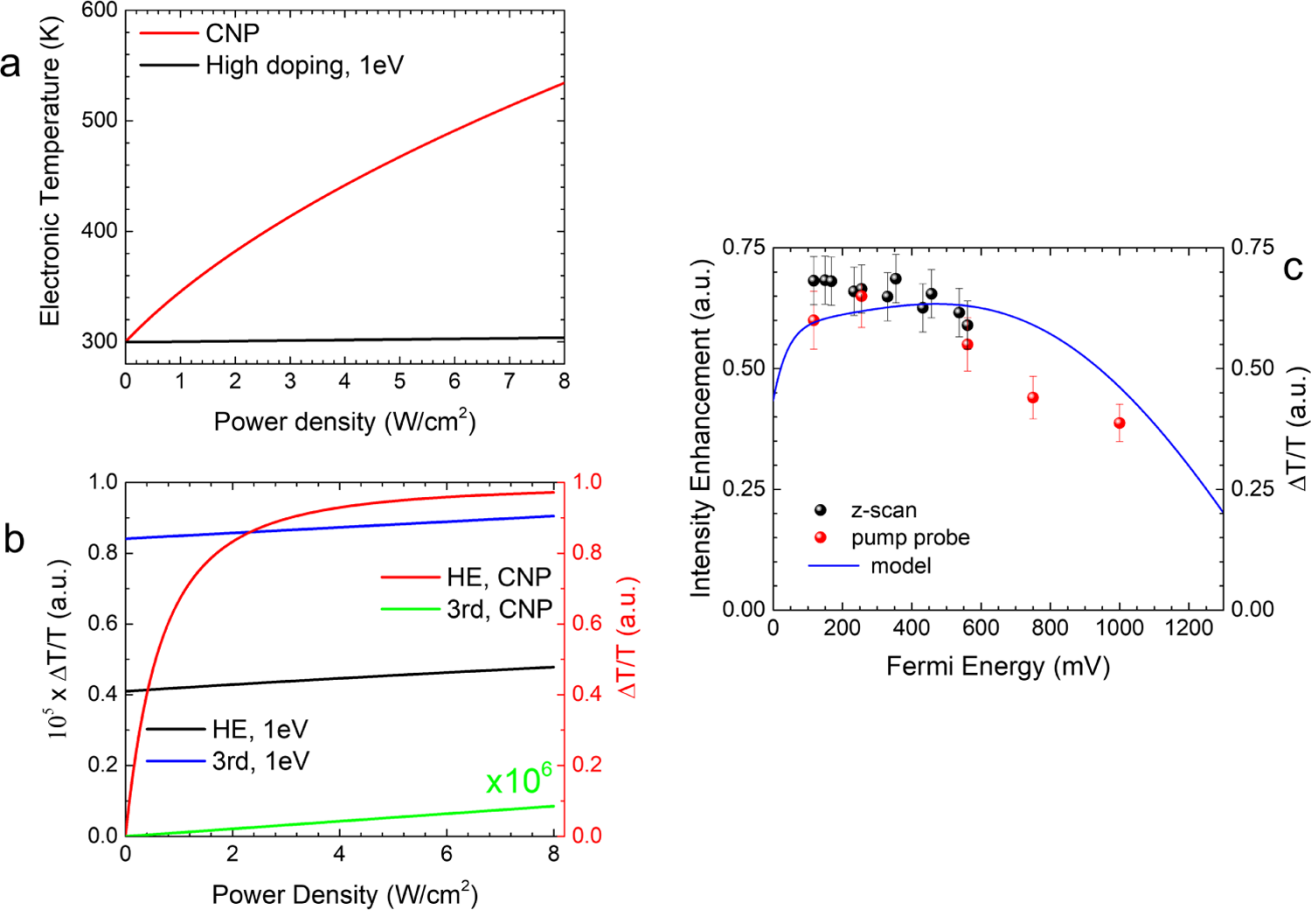
(a) *T*_e_ calculated by combining eqs 4 and 5 under a steady-state heating process, as a function of the impinging power density for a SLG film at low (CNP, red line) and high (*E*_F_ = 1 eV, black line) doping. (b) Δ*T*/*T* curves plotted as a function of the power density at low (CNP, right red axis) and high (*E*_F_ = 1 eV, black left axis) doping, calculated from eq 6 with the thermal conductivity of eq 2 at *T* = *T*_e_ (red and black), and from eq 11 with the 3rd-order field-dependent conductivity of eqs 7 and 8 (blue and green), respectively. (c) Comparison between the measured intensity enhancement (right axis) at a fixed fluence from *z*-scan (black dots) and pump and probe (red dots) experiments and calculated total Δ*T*/*T* (left axis, blue line) as a function of *E*_F_.

*T*_e_ at the steady excitation state can be written as^25^
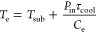
4where *T*_sub_ is the temperature at equilibrium (which approaches room temperature^25^), *P*_in_ is the excitation power density, and τ_cool_ is the cooling time. *C*_e_, the SLG electronic heat capacity, depends on *E*_F_ and on the temperature itself.^12,13^ For moderate (CNP, 50 meV) to highly doped SLG (*E*_F_ = 1 eV), this can be expressed as^25,54,55^
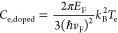
5Although we observe a clear Dirac point crossing in the IV transport measure (see Figure 1b) and in the intraband absorption-related transmittance (see Figure 1c), we retrieve a not-negligible residual conductivity at the CNP, resulting in a residual doping of ∼10^12^ cm^–2^, corresponding to *E*_F_ ∼ 110 meV. Therefore, the assumption *E*_F_ > *k*_b_*T*_e_ for the validity of eq 5 (ref^25^) is essentially always satisfied. Hot electron cooling typically occurs via electron–optical phonon through a biexponential decay,^53^ with a subpicosecond initial decay related to direct coupling to optical phonons, and a few-picosecond decay due to the hot-phonon bottleneck.^56^ It is also influenced by the dielectric environment and by disorder.^57,58^ An accurate estimate of this parameter would require ultrafast pump and probe experiments. Here, we use τ_cool_ to describe the hot electron dynamics in a steady-state excitation, in agreement with previous reports on SLG on quartz,^17,23,59^ and by combining eqs 4 and 5, we calculate the *T*_e_ dependence from the power density, shown in Figure 4a, for the two experimental extreme cases: at CNP (*E*_F_ = 50 meV) and high doping (*E*_F_ = 1 eV). In order to maximize the temperate increase, it is convenient to operate at low *E*_F_ < 50 meV and, more generally, under low *C*_e_ ∼ 0.17 J/(m^2^·K).

Our experimental observations (absorption, reflection, and transmission) can be described by the complex conductivity of hot electrons. The temperature dependence of the intraband conductivity is accounted in eq 2, by setting *T* = *T*_e_.^60,61^ The transmission variation is defined as:^25,62,63^
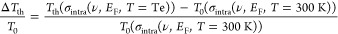
6where *T*_0_ is the equilibrium transmittance calculated from eq 1 by using the linear intraband conductivity of eq 2 at room temperature (300 K), and *T*_th_ is the nonlinear transmittance due to thermal effects, calculated by considering the hot electron temperature *T*_e_ in the optical conductivity of eq 2. Furthermore, in the presence of an intense beam, the SLG nonlinear response is affected by a field-dependent conductivity. For our SLG device pumped with a monochromatic, linearly polarized, normally incident radiation beam, we consider the nonlinear response beyond thermal effects up to the 3rd order. Since the SLG owns a centrosymmetric structure, even-order processes are forbidden. The QCL pump induces a polarization current oscillating in the same direction as the incoming field at the frequency of the incident wave υ (Kerr effect).

The field-dependent conductivity is then:
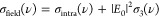
7where *E*_0_ is the electric field amplitude, defined as .

By combining eq 7 with the graphene displacement current density, *J*_gra_ (ν) = σ_field_ (ν) × *E*_0_ (ν), one can see that the nonlinear term of the absorption coefficient (see eq 6) scales as the 3rd-order power of the electric field, meaning that the nonlinear response of the graphene is a 3rd-order process.

The field-dependent 3rd-order term of the conductivity, σ_3_, is^64^:
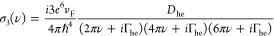
8where the *E*_F_-dependent parameters *D*_he_ and Γ_he_ are the hot electron Drude weight and scattering rate, respectively, defined as^64^
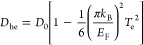
9
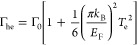
10The Drude weight and scattering time show a 2nd-order hot electron temperature dependence, meaning that  is still affected by hot electrons.

Similarly to the approach adopted in eq 6, we then calculate the transmission variation induced only by the field-driven 3rd-order nonlinearity by combining eqs 9 and 10, in the case of low and high doping (Figure 4b). Then, we compare it with the one retrieved by considering only the thermal effects in eq 6:
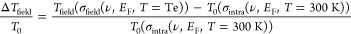
11where *T*_field_ is the transmittance calculated from eq 1 with σ = σ_field_.

In both cases, the total conductivity is reduced, hence quenching the absorption, in agreement with experiments. The 3rd-order nonlinear efficiency is 10^–5^–10^–6^ at the highest QCL power available and in the whole range of doping levels (see Figure 4b), making the observation of any possible third harmonic generation (THG) signal well below the noise level of the present experimental system. As expected, the hot electron temperature variation is maximized at low *E*_F_ = 50 meV, where a lower number of carriers are available to share the excess thermal energy. Conversely, the field-driven 3rd-order nonlinearity is favored at high *E*_F_ = 1 eV.^65^

Finally, in Figure 4c, we compare the experimental data, i.e., the transmission enhancement retrieved from *z*-scan and pump and probe experiments at different *V*_G_, with the total Δ*T*/*T*, calculated by using the same method of eqs 6 and 11, setting the total conductivity as σ = σ_field_ + σ_th_, and by considering the peak fluence in the *z*-scan (Figure 2) and in the pump and probe experiments (Figure 3).

We observe a strong saturation of the transmittance enhancement at the highest power densities for the lowest doping levels probed, while the model predicts a rapid decrease of Δ*T*/*T* with *E*_F_ at very low *E*_F_. This behavior is related to the accuracy of the complex conductivity calculation in the regime close to the CNP, not accounting for the minimum conductivity of SLG at the Dirac point.^53^ At very low *E*_F_ = 50 meV, the intraband cooling channel returns available, and the corresponding term in the optical conductivity should be taken into account. The excellent agreement at moderate *E*_F_ ∼ 150–600 meV and high *E*_F_ = 1 eV confirms the fundamental role of hot electrons in governing the nonlinear response of SLG in the steady excitation state.

## Conclusions

We show that the doped SLG pumped with QCLs at >3 THz has a nonlinear behavior. The retrieved nonlinearity is dominated by hot carriers, with a 3rd-order field-driven nonlinear contribution that affects the optical conductivity at high *E*_F_ > 0.8 eV. This provides a path for THG at >9 THz, a frequency range unexplored so far, due to the lack of compact sources operating in the 25–60 μm range. To overcome challenges imposed by the small Drude weight of SLG conductivity at 3 THz, one could use patterned SLG ribbons^66^ in order to shift and enhance the Drude conductivity at the QCL pump frequency, which will in turn enable higher conversion efficiencies for THG. Our findings also pave the way for exploiting SLG in applications that require control of its nonlinear behavior, as efficient nonlinear THz modulators, shutters, and ultrafast switches,^3^ whose speeds are ultimately limited by the carrier cooling time of a few picoseconds (e.g., few hundred GHz bandwidths) of interest for ultrahigh-speed communications.

## Materials and Methods

### SLG Growth, Transfer, and Raman Characterization

SLG is grown by CVD^67^ and wet-transferred on a *z*-cut quartz substrate. Both the as-grown and transferred SLGs are characterized by Raman spectroscopy (see the Supporting Information, SI). A statistical analysis on the as-grown SLG on Cu and on transferred SLG on *z*-cut quartz is performed to estimate *E*_F_ and defect density. From the Raman study, we extracted p doping with *E*_F_ = 250 ± 70 meV,^44,68^ which corresponds to n = 4.8 ± 2.6 × 10^12^ cm^–2^ ^44,68^ and a defect density *n*_D_ = (3.0 ± 1.8) × 10^9^ cm^–2^.^69^

### SLG-Gated Modulator Fabrication

The SLG-gated device modulator (see the SI) is realized as follows: CVD SLG is transferred on *z*-cut quartz, and source, drain, and coplanar gate metal electrodes (5 nm Cr/50 nm Au) are deposited using a shadow mask. The electrically switchable optical window has an area of 6 × 6 mm^2^. The modulator stack is sealed, positioning a 0.1 mm thick quartz substrate on top of the structure (see Figure 1). Finally, the SLG active area is then covered by the ionic liquid (*N*,*N*-diethyl-*N*-methyl-*N*-(2-methoxyethyl ammonium-bis-trifluoromethanesulfonyl imide—99.9%)).

### QCL Fabrication

The single-plasmon THz QCL, emitting at 2.68 THz (λ = 111 μm), employed for the experiments in Figure 2, relies on a bound-to-continuum active region design. It is fabricated through a combination of optical lithography and metal deposition, with a surface-plasmon waveguide onto a semi-insulating 150 μm thick GaAs substrate. 12 μm wide (80–100 nm GeAu/Au) stripes are introduced on the two edges of the ridge to improve the electrical contact. The 10 μm thick GaAa/AlGaAs active region comprises 120 repetitions. This is embedded between two heavily Si-doped GaAs layers defining the bottom (500 nm thick, *n* = 3 × 10^18^ cm^–3^) and top (200 nm thick, *n* = 5 × 10^18^ cm^–3^) contacts. Laser bars are 2 mm long and 180 μm wide.

The high-power THz QCL employed for the experiments described in Figure 3 is based on a hybrid active region design (bound-to-continuum-resonant photon) operating at ∼3.2 THz, with a 25 μm active region height. The device is 3 mm long with widths of 437 μm and is fabricated in a single-plasmon optical waveguide. 5 nm thick, 40 μm wide Ni side-absorbers are introduced on the two edges of the ridge to increase the difference in losses between the fundamental and higher-order transverse modes and achieve full suppression of higher-order competing modes. An overlap of 3 μm between Ni side-absorber and upper Au overlayer, 150 nm thick, is set by design. The 700 nm heavily Si-doped (5 × 10^18^ cm^–3^) GaAs top contact layer lies between the active region and substrate.^70^

## Supporting Information Available

Saturation absorption (S1); Raman characterization of SLG (S2); fabrication of SLG-gated modulator (S3); and QCL fabrication (S4) (PDF)
