# Supplementary material for: Electrically Tunable Nonlinearity at 3.2 Terahertz in Single-Layer Graphene
Source: ACS Photonics. 2023 Aug 14;10(9):3171–80. doi: 10.1021/acsphotonics.3c00543 (PMC10515698; doi:10.1021/acsphotonics.3c00543)
Supplement: Supplementary file 1 — ph3c00543_si_001.pdf [file ph3c00543_si_001.pdf]

Supporting Information

Electrically-tunable non-linearity at 3.2 terahertz in single layer graphene

Alessandra Di Gaspare,[1] Osman Balci,[2] Jincan Zhang,[2] Adil Meersha,[2] Sachin M. Shinde,[2] Lianhe Li,[3] A. Giles Davies,[3] Edmund H. Linfield,[3] Andrea C. Ferrari[2] and Miriam S. Vitiello[1*]

[1] NEST, CNR - Istituto Nanoscienze and Scuola Normale Superiore, Piazza San Silvestro 12, 56127, Pisa, Italy
[2]Cambridge Graphene Centre, University of Cambridge, Cambridge CB3 0FA, UK
[3] School of Electronic and Electrical Engineering, University of Leeds, Leeds LS2 9JT, UK

*miriam.vitiello@sns.it

S1. Saturation absorption

In a *z*-scan experiments, by assuming a nearly Gaussian profile of the optical beam impinging on the sample in the focal region, the absorption coefficient dependence on the beam intensity, A(I) is expressed as[1]:

$$A(I) = A_0 + \frac{A_s}{\left(1 + \frac{I}{I_S}\right)} \quad \text{(S1)}$$

where A_0 is the linear non-saturated absorption, A_s is the saturated component of the absorption, I is the optical intensity (in Wcm-2) and I_s is the saturation intensity.
In the transmission-mode configuration, employed in the present work, we can relate eq.S1 with the measured transmittance, by assuming T~1-A, (i.e. by neglecting the reflection term in the quasi transparent quartz sample). Therefore:

$$T(I) \sim 1 - A(I) = 1 - A_0 - \frac{A_s}{\left(1 + \frac{I}{I_S}\right)} = T_0 + \frac{T_s}{\left(1 + \frac{I}{I_S}\right)} \quad \text{(S2)}$$

where we have set $T_0 = 1 - A_0$ and $T_S = -A_S$, meaning that that the observed transmission enhancement coincides with the saturated component of the absorption.

S2. Raman characterization of SLG

As grown and transferred SLG are both characterized by Raman spectroscopy with a Renishaw InVia Raman spectrometer at 514 nm using 100x objective with power on the sample < 0.5 mW to exclude heating effects. A statistical analysis of five spectra on as-grown SLG on Cu, thirty spectra on transferred SLG on z-cut quartz is performed to estimate E_F and defect density. The errors are calculated from the standard deviation across different spectra, the spectrometer resolution (~1 cm^{-1}) and the uncertainty associated with the different methods to estimate E_F from Pos(G), FWHM(G), I(2D)/I(G), A(2D)/A(G) and Pos(2D) and the defect density from I(D)/I(G). The Raman Spectrum of as grown SLG on Cu is shown in **Fig. S1**, after Cu photoluminescence removal[2]. The 2D peak is a single Lorentzian with FWHM(2D) = 23 ± 3 cm^{-1}, signature of SLG[3]. The position of the G peak, Pos(G), is 1586 ± 2 cm^{-1}, with FWHM(G) = 15 ± 1 cm^{-1}. The 2D peak position, Pos(2D), is 2705 ± 5 cm^{-1}, while the 2D to G peak intensity and area ratios, I(2D)/I(G) and A(2D)/A(G), are 4.3 ± 0.2 and 6.7 ± 0.7. No D peak is observed, indicating negligible Raman active defecs[4,5]. The Raman spectrum of SLG transferred on z-cut quartz substrate is in **Fig. S1**. The 2D peak retains its single-Lorentzian line shape with FWHM(2D) = 27 ± 1 cm^{-1}. Pos(G) = 1591±2 cm^{-1}, FWHM(G) = 10 ± 2 cm^{-1}, Pos(2D) = 2691 ± 2 cm^{-1}, I(2D)/I(G) = 2.5 ± 0.5 and A(2D)/A(G)= 6.7 ± 0.6 indicating a p doping with E_F = 250 ± 70 meV[6,7], which corresponds to n=4.8±2.6x10^{12} cm^{-2} [6,7]. I(D)/I(G) = 0.01 ± 0.01 corresponds to a defect density n_D = (3.0 ± 1.8) x 10^9 cm^{-2} [8] for 2.41eV excitation.

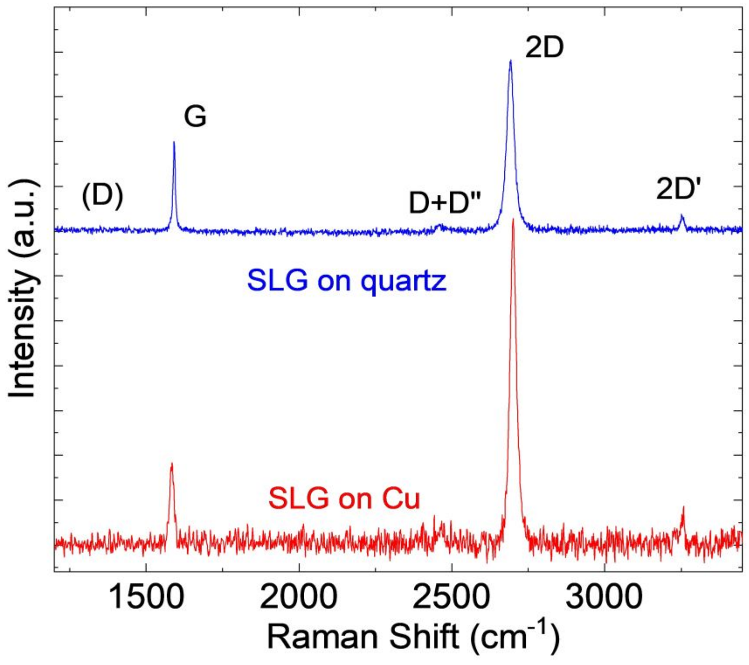

Figure S1: 514 nm Raman spectra of as-grown SLG on Cu (red) and transferred SLG on quartz (blue).

S3. Fabrication of SLG gated modulator

The SLG gated device modulator is schematically illustrated in Fig.S2.

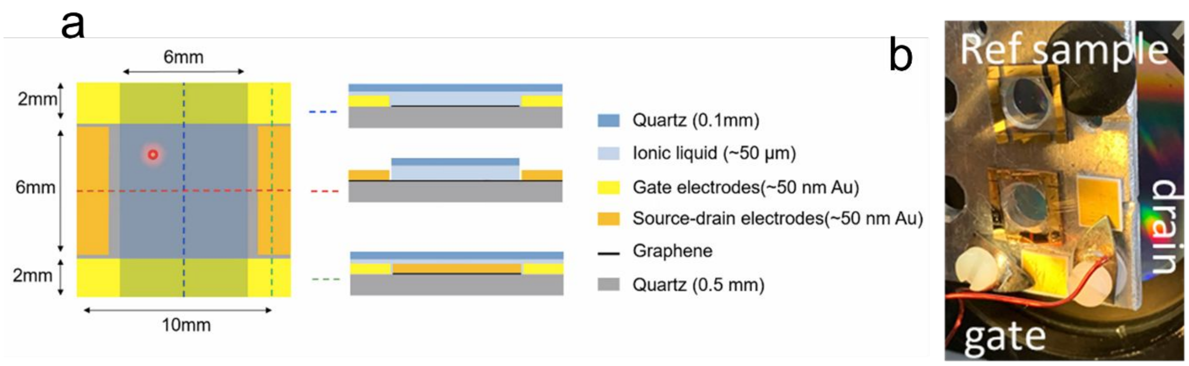

Figure S2: (a) Schematic layout of SLG device. (b) Photograph of device mounted on mechanical holder used for the optical experiment. The reference sample is mounted on the same holder, enabling the data optical measurement in the same experiment by moving the vertical axis at normal incidence.

S4. QCL fabrication

Fig. S3 shows the current-voltage-light characteristic of the high-power THz QCL employed for the pump and probe experiments described in Fig. 3 of the main article, is based on an hybrid active region design (bound to continuum-resonant photon) operating at ~3.25 THz, with a 25 μm active region height. The device is 3 mm-long, with widths of 437 μm and is fabricated in a single plasmon optical waveguide.

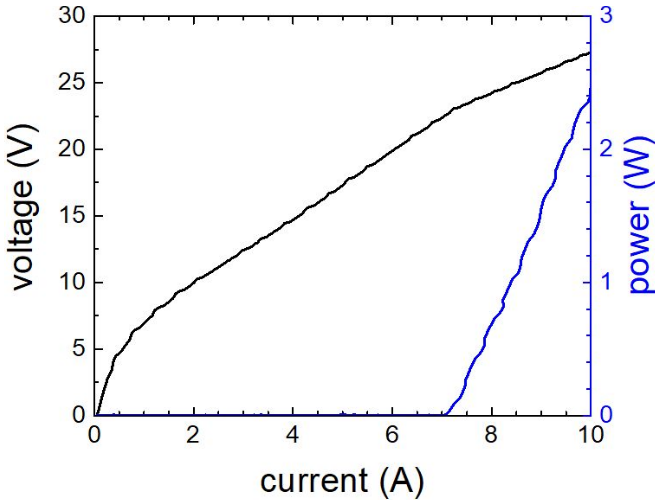

Figure S3: current–voltage (I–V) and current density–optical power (I–L) characteristics of single plasmon QCL as pump THz beam in the setup of Fig.3, measured at a heat-sink temperature of 18 K while driving the lasers in pulsed mode with a pulse width of 1 μs and a repetition rate of 10 kHz (1%-duty cycle) in vacuum.

References

(1) Bianchi, V.; Carey, T.; Viti, L.; Li, L.; Linfield, E. H.; Davies, A. G.; Tredicucci, A.; Yoon, D.; Karagiannidis, P. G.; Lombardi, L.; Tomarchio, F.; Ferrari, A. C.; Torrisi, F.; Vitiello, M. S. Terahertz Saturable Absorbers from Liquid Phase Exfoliation of Graphite. *Nat. Commun.* **2017**, *8* (1), 15763. https://doi.org/10.1038/ncomms15763.

(2) Lagatsky, A. A.; Sun, Z.; Kulmala, T. S.; Sundaram, R. S.; Milana, S.; Torrisi, F.; Antipov, O. L.; Lee, Y.; Ahn, J. H.; Brown, C. T. A.; Sibbett, W.; Ferrari, A. C. 2 Mm Solid-State Laser Mode-Locked by Single-Layer Graphene. *Appl. Phys. Lett.* **2013**, *102* (1), 13113. https://doi.org/10.1063/1.4773990.

(3) Ferrari, A. C.; Meyer, J. C.; Scardaci, V.; Casiraghi, C.; Lazzeri, M.; Mauri, F.; Piscanec, S.; Jiang, D.; Novoselov, K. S.; Roth, S.; Geim, A. K. Raman Spectrum of Graphene and Graphene Layers. *Phys. Rev. Lett.* **2006**, *97* (18), 187401. https://doi.org/10.1103/PhysRevLett.97.187401.

(4) Ferrari, A. C.; Robertson, J. Interpretation of Raman Spectra of Disordered and Amorphous Carbon. *Phys. Rev. B* **2000**, *61* (20), 14095–14107. https://doi.org/10.1103/PhysRevB.61.14095.

(5) Ferrari, A. C.; Basko, D. M. Raman Spectroscopy as a Versatile Tool for Studying the Properties of Graphene. *Nat. Nanotechnol.* **2013**, *8* (4), 235–246. https://doi.org/10.1038/nnano.2013.46.

(6) Das, A.; Pisana, S.; Chakraborty, B.; Piscanec, S.; Saha, S. K.; Waghmare, U. V; Novoselov, K. S.; Krishnamurthy, H. R.; Geim, A. K.; Ferrari, A. C.; Sood, A. K. Monitoring Dopants by Raman Scattering in an

Electrochemically Top-Gated Graphene Transistor. *Nat. Nanotechnol.* **2008**, *3* (4), 210–215. https://doi.org/10.1038/nnano.2008.67.

(7) Basko, D. M.; Piscanec, S.; Ferrari, A. C. Electron-Electron Interactions and Doping Dependence of the Two-Phonon Raman Intensity in Graphene. *Phys. Rev. B* **2009**, *80* (16), 165413. https://doi.org/10.1103/PhysRevB.80.165413.

(8) Bruna, M.; Ott, A. K.; Ijäs, M.; Yoon, D.; Sassi, U.; Ferrari, A. C. Doping Dependence of the Raman Spectrum of Defected Graphene. *ACS Nano* **2014**, *8* (7), 7432–7441. https://doi.org/10.1021/nn502676g.
